# Prevalence of *Eimeria* spp. in goats from northern Paraná, Brazil

**DOI:** 10.1590/S1984-29612025025

**Published:** 2025-06-13

**Authors:** Carla Adriele Rodrigues, Jonas Correia de Araujo, Jorgeana Guadanhini Negrizolli, Maria Júlia Papin Filadelfo, Mateus Siqueira Pyles, Ana Carolina Cavalieri, Luiz Daniel de Barros, Fernando de Souza Rodrigues, João Luis Garcia

**Affiliations:** 1 Laboratório de Protozoologia, Departamento de Medicina Veterinária Preventiva, Centro de Ciências Agrárias, Universidade Estadual de Londrina – UEL, Londrina, PR, Brasil; 2 Laboratório de Parasitologia Veterinária e Doenças Parasitárias, Departamento de Medicina Veterinária, Universidade Federal de Lavras – UFLA, Lavras, MG, Brasil

**Keywords:** Coccidiosis, caprine, Apicomplexa, Eimeriidae, Paraná, Brazil, Coccidiose, caprinos, Apicomplexa, Eimeriidae, Paraná, Brasil

## Abstract

This study aimed to evaluate the presence, identify *Eimeria* species, and epidemiological aspects associated with the infection in goat herds in northern Paraná, Brazil. A total of 384 fecal samples were collected from goats of different breeds, ages, and sexes from eight farms. An epidemiological questionnaire about rearing system (semi-intensive or intensive), age of the animals (up to 6 months or over 6 months), and type of floor in the pen (dirt or slatted) was used to evaluate the epidemiological aspects associated with *Eimeria* spp. infection. The McMaster technique was used to count *Eimeria* spp. oocysts. After oocyst counting, the samples were separated for sporulation and species identification. In total, 82.3% (316/384) of the samples showed positive results. For OPG, 37% of the animals exhibited counts >1,000 OPG (max.63600 OPG), whereas 63% had counts <1,000 OPG. Animals from semi-intensive production systems had high oocyst counts (mean-2139 OPG). Goats raised in pens with slatted floors (mean-1158 OPG) have lower average oocyst counts than those raised in facilities with dirt floors (mean-2714 OPG). Eight *Eimeria* species were identified *E. arloingi* (23.9%), *E. apsheronica* (19.7%), *E. ninakohlyakimovae* (14.3%), *E. alijevi* (12%), *E. caprina* (10%), *E. jolchijevi* (9%), *E. hirci* (6.9%) and *E. christenseni* (4.2%). This study showed a high prevalence of *Eimeria* spp. in goats in northern Paraná, Brazil.

## Resumo

O objetivo do estudo foi avaliar a presença, identificar espécies de *Eimeria* e aspectos epidemiológicos associados à infecção em rebanhos caprinos do Norte do Paraná, Brasil. Foram coletadas 384 amostras fecais de caprinos de diferentes raças, idades e sexos, de oito fazendas. Um questionário epidemiológico sobre o sistema de criação (semi-intensivo e intensivo), idade dos animais (até 6 meses e acima dessa idade) e o tipo de solo do curral (terra ou ripado) foi aplicado para avaliar aspectos epidemiológicos associados à infecção por *Eimeria* spp. A técnica de McMaster foi realizada para contagem de oocistos de *Eimeria* spp. Após contagem, as amostras foram separadas para esporulação e identificação das espécies. No total, 82,3% (316/384) apresentaram resultados positivos. Destes animais, 37% apresentaram contagens >1.000 OPG (máx. 63600OPG), enquanto 63% apresentaram contagens <1.000 OPG. Animais em sistemas semi-intensivos apresentaram contagens elevadas de oocistos (média-2139 OPG). Caprinos criados em baias com piso ripado (média-1158 OPG) apresentaram contagem média de oocistos menor do que cabras em baias com piso de terra (média-2714). Oito espécies de *Eimeria* foram identificadas *E. arloingi* (23,9%), *E. apsheronica* (19,7%), *E. ninakohlyakimovae* (14,3%), *E. alijevi* (12%), *E. caprina* (10%), *E. jolchijevi* (9%), *E. hirci* (6,9%) e *E. christenseni* (4,2%). Este estudo mostrou alta prevalência de *Eimeria* spp. em caprinos do norte do Paraná, Brasil.

## Introduction

Goat farming is a livestock activity that is increasingly expanding in Brazil and in the state of Paraná. According to data from the Brazilian Institute of Geography and Statistics (IBGE), in 2021 the Brazilian goat population was 11,923,630 million animals, with 80,972 thousand in Paraná (IBGE, 2021).

Eimeriosis, or coccidiosis, is a disease caused by infection with protozoa of the genus *Eimeria*. Infection occurs through the ingestion of sporulated oocysts in ruminants and mainly infects intestinal cells (Keeton & Navarre, 2018; Bangoura & Bardsley, 2020; Andrews, 2013; Chartier & Paraud, 2012). Most clinical cases are observed in juveniles; however, adult animals can shed oocysts that contaminate the environment (Andrews, 2013; Chartier & Paraud, 2012; Bangoura & Bardsley 2020; Koudela & Boková, 1998; Palomino-Guerrera et al., 2024). Kids between 2 and 4 months of age excrete oocysts heavily and can develop diarrhea and dehydration, which in some cases can be fatal (Koudela & Boková, 1998; Andrews, 2013; Chartier & Paraud, 2012; Ruiz et al., 2006; Wang et al., 2010; Rufino-Moya et al., 2024). Subclinical infections reduce productivity through weight loss and adverse effects on feed conversion and long-term performance (Keeton & Navarre, 2018; Abo-Shehada & Abo-Farieha, 2003; Jalila et al., 1998).

Various *Eimeria* species can infect goats; however, not all are associated with clinical disease. In goats, *E. ninakohlykimovae, E. arloingi* and *E. caprina* are the most pathogenic *Eimeria* species (Cavalcante et al., 2012; Chartier & Paraud, 2012; Andrews, 2013; Levine, 1985; Dai et al., 2006; Bangoura & Bardsley, 2020; Ruiz & Molina, 2019).

In Brazil, *Eimeria* spp. infection in goats is distributed, with reports of infections in different regions of the country (Carvalho et al., 2023; Macedo et al., 2020; Cavalcante et al., 2012); however, there are few studies in South of Brazil (Radavelli et al., 2014). Epidemiological studies of coccidiosis can support developing and implementing disease control programs (Carvalho et al., 2023).

Since, no report is available from northern Parana, Brazil, and the clinical effects, and economic costs cause by the protozoa, the present study was designed to evaluate the presence and identify the species of *Eimeria* and determine aspects of the epidemiology of coccidiosis in goat herds in the northern area of the State of Paraná.

## Material and Methods

**Figure 1:**
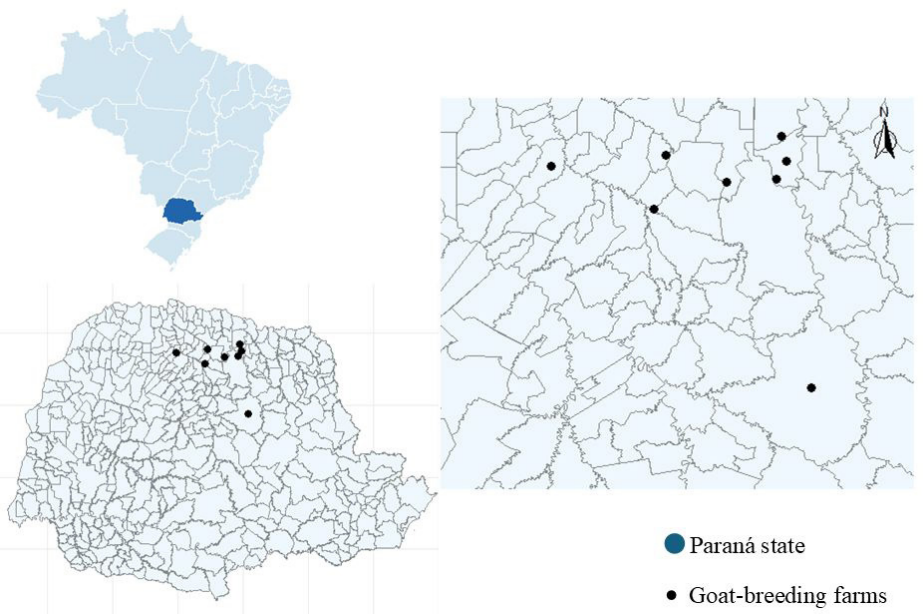
Map showing the goat breeding farms sampled from northern Paraná, Brazil.

The sample calculation was carried out using the EpiInfo® program, using a population of 80,972 thousand heads of goats in the state of Paraná (IBGE, 2021), an estimated prevalence of 50%, with an acceptable error of 5% (45-55%) and a confidence level of 95%. A total of 384 fecal samples were collected from goats of different breeds, ages, and sexes from eight goat-breeding farms (Figure 1) located in five counties in the northern region of the State of Paraná, Brazil (Apucarana, Londrina, Maringá, Ortigueira, and Paiçandu). All animals were not treated for eimeriosis.

The following epidemiological aspects of *Eimeria* spp. infection were evaluated: rearing system (semi-intensive or intensive), age of the animals (< 6 months or > 6 months), and type of soil in the pen (dirt or slatted).

Fecal samples were collected randomly and directly from the rectum, identified, and stored in a thermal box on ice until processing. In the laboratory, the McMaster technique was used to count *Eimeria* spp. oocysts per gram (OPG) of feces with a sensitivity of 50 (Gordon & Whitlock, 1939).

After oocyst counting, the samples were separated for sporulation and species identification. For sporulation, samples were separated according to the age of the animals (< 6 months and > 6 months). This mixture was filtered through distilled water and potassium dichromate (K_2_Cr_2_O_7_) was added at a concentration of 2% at a 1:1 ratio with distilled water. This material was maintained for 10 d under forced aeration. After sporulation, the sporulated oocysts were stored in Falcon tubes at 8 °C until identification.

For *Eimeria* species identification, oocysts and sporocysts were measured using an Olympus BX43 optical microscope (Olympus Corporation, Tokyo, Japan) at 100× magnification coupled to a digital camera (Q-Color3™ imaging system, Olympus Corporation) for photographic documentation of the oocysts. The digital images obtained were analyzed using standard CellSens software, version 1.15 2016 (Olympus Corporation). The morphometric variables measured were the length, width, and thickness of the walls of the sporulated oocysts and sporocysts. The morphological characteristics considered for identification were shape, color, the presence or absence of a micropyle, and the shape and size of the sporocysts (Eckert et al., 1995; Ruiz & Molina, 2019).

Statistical analysis was performed using R (R Core Team R, 2023) and a p-value ≤0.05 was set as the cut-off for statistical significance. OPG was not normally distributed (Kolmogorov–Smirnov test, p < 0.05, data not shown). Therefore, the nonparametric Mann–Whitney U test was used to compare the mean OPG between the risk factors analyzed (age, type of floor, and rearing system).

## Results

**Figure 2:**
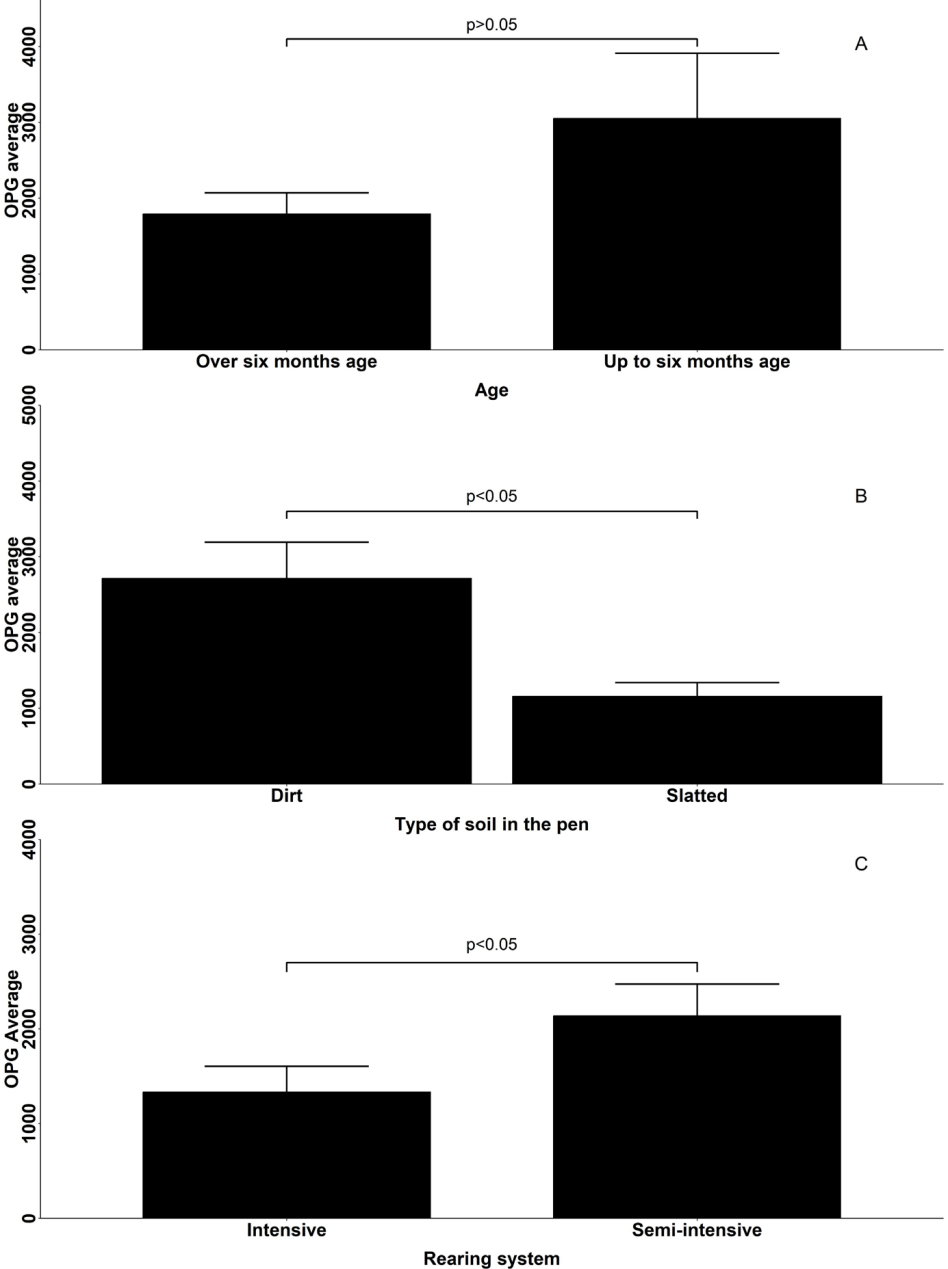
Oocysts per gram of feces (OPG), averaged values from goats from northern Paraná, Brazil according to different variables. (A) age; (B) type of soil; (C) rearing system.

In total, 82.3% (316/384) of the samples showed positive results. For OPG, 37% (142/384) of the animals exhibited counts >1,000 OPG, whereas 63% (242/384) had counts <1,000 OPG. Of the 384 goats, 13% (50/384) were up to six months of age, and the average OPG of these animals was 2,776, while the other 87% over six months (334/384) had an average OPG of 1,825. However, there were no significant differences between the OPG of animals of different ages (Figure 2A).

Of the eight farms, five had semi-intensive farming practices and three had intensive farming practices. Five of the properties had slatted floors, whereas the remaining three have dirt floors. Among animals kept on dirt floors (199/384), the average OPG was 2,684; among those on slatted floors (185/384), it was 1,158 (p<0.05) (Figure 2B). Most of the animals were maintained in a semi-intensive system (297/384). The average OPG of the animals under semi-intensive and intensive rearing was 2,129 and 1,333, respectively (p<0.05) (Figure 2C).

**Figure 3:**
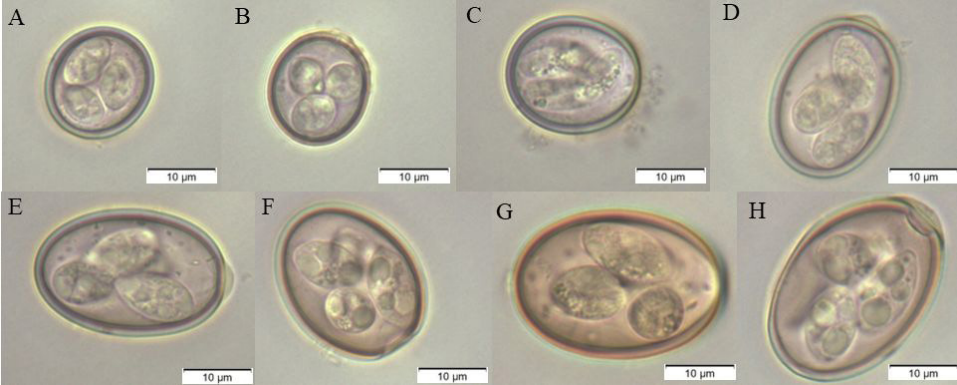
*Eimeria* spp. identified in goats from northern Paraná, Brazil. (A) *Eimeria alijevi*; (B) *Eimeria hirci*; (C) *Eimeria ninakohlyakimovae*; (D) *Eimeria arloingi*; (E) *Eimeria jolchijevi*; (F) *Eimeria aspheronica*; (G) *Eimeria caprina*; (H) *Eimeria christenseni*.

Eight species of *Eimeria* were identified in this study (Figure 3). The most prevalent species was *E. arloingi*. In goats over six months old, the most prevalent species was *E. arloingi* (24.8%), followed by *E. aspheronica* (16.8%), and *E. ninakohlyakimovae* (15%). Among those up to six months old, the most common species was *E. arloingi* (21.7%), followed by *E. alijevi* (15.7%), and *E. caprina* (15%) (Table 1).

**Table 1:** Frequencies of Eimeria species in goats from northern Paraná, Brazil.

	**Age**			
**Species**	**Over six months of age**		**Up to six months of age**	
	n	%	n	%
E. arloingi*	177	24.8%	65	21.7%
*E. apsheronica*	152	21.3%	48	16%
*E. ninakohlyakimovae* *	107	15%	38	12.7%
*E. alijevi*	75	10.5%	47	15.7%
*E. caprina*	56	7.8%	45	15%
*E. jolchijevi*	72	10.1%	19	6.3%
*E. hirci*	51	7.1%	19	6.3%
*E. christenseni*	24	3.4%	19	6.3%
**TOTAL**	**714**	**100%**	**300**	**100%**

* Pathogenic species.

## Discussion

In the present work, we found that 82.3% of animals were infected with *Eimeria* spp. Similar results were found in previous studies in Brazil, which showed a prevalence of 73.13% and 99.4% in positive samples (Carvalho et al., 2023; Cavalcante et al., 2012; Macedo et al., 2020; Hassum & Menezes, 2005). In other countries, the prevalence was also high: in Southwest Montana in the United States, a prevalence of 97.2% was described (Penzhorn et al., 1994); in Peru 86.22% (Palomino-Guerrera et al., 2024), in Central Europe (Ukraine and Poland), 66.7% in Thailand (Rerkyusuke et al., 2024), 74% (Balicka-Ramisz et al., 2012); in Tanzania, 94.7% (Kusiluka et al., 1996); and in Shaanxi Province, Northwest China, it was 97.3% (Zhao et al., 2012). However, the prevalence may be lower, with results between 17.3% and 41.3% (Ahid et al., 2008; Koinari et al., 2013; Das et al., 2018). The difference in prevalence may occur because of several factors, such as the number of sporulated oocysts ingested, multiplication of the parasite in the host, population density, production system in which the animals are raised, age, post-weaning period, stressful conditions, failure of immunity, and environmental factors: types of facilities and facility floors, climate, temperature, high humidity in the facilities, and lack of cleanliness in the stalls (Balicka-Ramisz, 1999; Harper & Penzhorn, 1999; Ruiz et al., 2006; Zvinorova et al., 2016; Molina & Ruiz, 2019).

In this study, kids up to 6 months of age were more frequently infected and had a higher average excretion of oocysts than goats older than 6 months; however, the difference was not statistically significant. The lack of a statistical difference may be due to the small number of animals up to six months old. Age is an important factor in *Eimeria* infections of goats. The high susceptibility of kids is related to their weaning period. Generally, in the first weeks of life, suckling kids become infected by ingesting oocysts attached to goat teats. From 4 weeks to 6 months of age, kids start to excrete large quantities of oocysts into the environment, making it a high-risk period for environmental contamination and infecting other animals (Kanyari, 1993; Jalila et al., 1998; Chartier & Paraud, 2012; Guedes et al., 2024; Rufino-Moya et al., 2024).

Goats over 6 months of age, despite having a high average OPG, had a lower count than kids (<6 months). Continuous contact with the parasite allows for the development of acquired immunity, and adult goats have significantly lower OPG levels than kids (Balicka-Ramisz et al., 2012; Rufino-Moya et al., 2024). Other studies have also shown a negative correlation between OPG levels and goat age (Kanyari, 1993).

A statistically significant difference in OPG was detected between the intensive (2,129 OPG) and semi-intensive (1,333 OPG) systems. Differences in OPG counts between production systems are probably related to facility hygiene and applied sanitary management (Ruiz et al., 2006). In intensive and semi-intensive production systems, high OPG counts can occur due to the high population density of these systems, promoting the spread of infection in the herd (Sharma et al., 2017). Furthermore, studies that evaluated the influence of management factors on infections by *Eimeria* showed that the prevalence and intensity of infection were similar regardless of the production system (Ruiz et al., 2006).

Goats raised in pens with slatted floors had significantly lower average OPG counts than those raised in facilities with dirt floors. The slatted floor may have favored lower counts because of the reduced accumulation of feces containing oocysts in the facilities. In contrast, a dirt floor (with/without a bed) depends more on hygiene, as it accumulates more feces with oocysts in the environment, promoting higher oocyst counts (Macedo et al., 2020).

Most samples in this study were positive for different *Eimeria* spp., including pathogenic (i.e., *E. arloingi* and *E. ninakohlyakimovae*) and non-pathogenic species. The most frequent species was *Eimeria arloingi* (24%), which is generally most commonly found in intensive production systems (Ruiz et al., 2006). The other most frequent species were *E. apsheronica* (18%) and *E. ninakohlyakimovae* (14%), with emphasis on *E. ninakohlyakimova*e, which is the most pathogenic species in goats (Dai et al., 2006; García-Álvarez et al., 2023; Koudela & Boková, 1998; Ruiz et al., 2006). Pathogenicity is related to the replication site; in general, the most pathogenic species are *E. ninakohlyakimovae* and *E. arloingi,* which pass through the intestinal epithelium and invade the endothelial cells of the central lymphatic capillaries of the intestinal villi. Once in the endothelial cells, large macroschizonts are formed and require prolonged replication times. This process can lead to severe cellular damage in the intestinal mucosa (Ruiz et al., 2010, 2013).

## Conclusion

Based on the results of this study, the prevalence of infection by *Eimeria* spp. in goats in the North of Paraná was high. Animals kept in semi-intensive systems and on dirt floors shed greater numbers of oocysts. The most prevalent species were *E. arloingi*, *E. apsheronica* and *E. ninakohlyakimovae.* This study demonstrates the importance of knowing the aspects of epidemiology and identification of species of *Eimeria* to understand different breeding systems and establish sanitary management to reduce *Eimeria* spp. infection.
